# D-Glucosamine Promotes Transfection Efficiency during Electroporation

**DOI:** 10.1155/2014/485867

**Published:** 2014-02-11

**Authors:** Kazunari Igawa, Naoko Ohara, Atsushi Kawakubo, Kouji Sugimoto, Kajiro Yanagiguchi, Takeshi Ikeda, Shizuka Yamada, Yoshihiko Hayashi

**Affiliations:** ^1^Department of Cariology, Nagasaki University School of Biomedical Sciences, Nagasaki 852-8588, Japan; ^2^Department of Conservative Dentistry, Okayama University Graduate School of Medicine, Dentistry and Pharmaceutical Sciences, Okayama 700-8558, Japan

## Abstract

D-Glucosamine is a useful medicament in various fields of medicine and dentistry. With respect to stability of the cell membrane, it has been reported that bradykinin-induced nociceptive responses are significantly suppressed by the direct application of D-glucosamine. Electroporation is usually used to effectively introduce foreign genes into tissue culture cells. Buffers for electroporation with or without D-glucosamine are used in experiments of transfection vectors. This is the first study to indirectly observe the stability and protection of the osteoblast membrane against both electric stress and gene uptake (the proton sponge hypothesis: osmotic rupture during endosomes prior to fusion with lysosomes) in electroporation with D-glucosamine application. The transfection efficiency was evaluated as the fluorescence intensity of the transfected green fluorescent protein (GFP) in the cultured cells (osteoblasts; NOS-1 cells). The transfection efficiency increased over 30% in the electroporation samples treated with D-glucosamine-supplemented buffer after one day. The membrane absorption of D-glucosamine is the primary mechanism of membrane stress induced by electric stress. This new function of D-glucosamine is useful and meaningful for developing more effective transformation procedures.

## 1. Introduction

D-Glucosamine is a useful medicament in various fields of medicine and dentistry [1, 2]. For example, it is an attractive candidate for adjunctive therapy in patients with arthritis due to both its chondroprotective actions and anti-inflammatory and wound healing effects achieved via the suppression of the neutrophil function and chemokine production [3]. D-Glucosamine also has a significant antipain effect in patients with osteoarthritis. Therefore, D-glucosamine is widely used in an attempt to suppress the pain associated with the disability of osteoarthritis [4]. Recently, our group reported that bradykinin-induced nociceptive responses were significantly suppressed by the direct application of D-glucosamine [5], suggesting that D-glucosamine has a direct effect in relieving pain by ensuring membrane stability. Furthermore, we previously reported that D-glucosamine hydrochloride promoted the lysosomal escape of quantum dots inside cells (unpublished data).

The aim of this study was to confirm the maintenance of cell membrane stability via the direct effects of D-glucosamine. We used the electroporation technique for gene transfection as an experimental model to investigate both membrane protection (stability) and gene protection. A buffer solution with or without D-glucosaminewas used in electroporation of the transfection vector. The transfection efficiency was quantitatively evaluated as the fluorescence intensity of transfected green fluorescent protein (GFP) in the cultured cells.

## 2. Materials and Methods

### 2.1. Preparation of Cultured Cells

Osteoblasts (NOS-1 cells [6]) derived from human osteosarcoma were used and cultured in *α*-MEM containing 10% FBS together with antibiotics. One day before electroporation, the NOS-1 cells were prepared to loose confluence in a 60 mm culture dish. After passage, the NOS-1 cells were seeded in a 35 mm glass-bottomed culture dish (number P35Gcol-1.5-14-C, MatTek Corp., MA, USA) at a density of 5 × 10^5^ cells (each group: three dishes), and *α*-MEM containing 10% FBS without antibiotics was used for culture in a humidified incubator at 37°C in an atmosphere of 5% CO_2_ and air.

### 2.2. Preparation of Chitosan Solution and Buffer

D-Glucosamine hydrochloride (molecular weight: approximately 215) was kindly supplied by Koyo Chemical Co., Ltd (Osaka, Japan). A 1% (W/V) stock solution was prepared by dissolving powder in 0.1% (V/V) acetic acid. The completely dissolved solution was neutralized to a pH value of 7.4, then sterilized with a 0.2 *μ*m filter. Electroporation buffer with or without 0.005% (W/V) D-glucosamine in *α*-MEM without either FBS or antibiotics was prepared for electroporation.

### 2.3. Procedures for Electroporation

**Figure 1:**
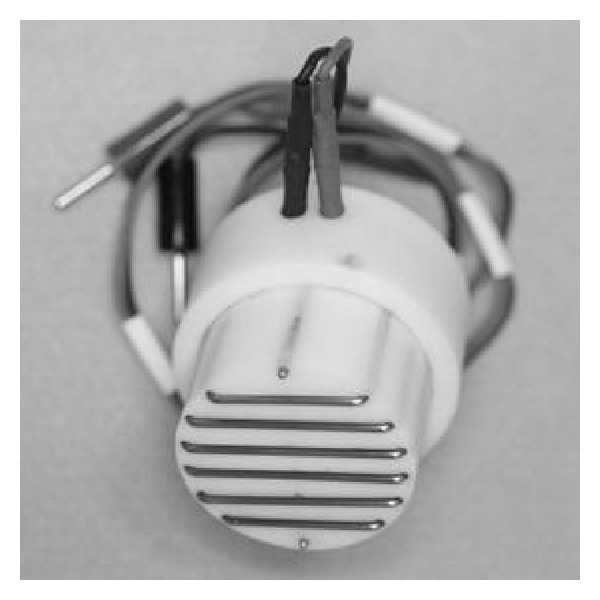
Wire type of electrode for electroporation.

Electroporation was performed using a wire type electrode (Figure 1) that was set vertical to the surface of the culture dish. One day after passage, the cells attached on the culture dish were treated using a commercial electroporator (CUY21B, Tokiwa Science Limited Company, Fukuoka, Japan), connected to the electrode. The pIRES2-EGFP vector (Clontech, Takara Bio Company, Shiga, Japan) was used as an expression plasmid during transfection. A total of 10 *μ*g/mL of the vector was prepared by dissolving the vector in the buffer. The treatment conditions consisted of five pulses of 120 V (effective voltage: 40–70 V, effective current: 40–4.4 A), each on: 5 ms and off: 95 ms, in 1 mL of electrode buffer with or without 0.005% (W/V) D-glucosamine.

### 2.4. Fluorescence Microscopy

The cells were cultured with *α*-MEM without either FBS or antibiotics for one day after electroporation and viewed using a confocal laser microscope (TCS SL, Leica Microsystems GmbH, Wetzlar, Germany) at a magnification of ×630.

### 2.5. Statistical Analysis

The number of GFP-positive cells was counted and converted to a percentage of the original cell number (100%) in three areas selected at random from the examined group supplemented with or without D-glucosamine. The statistical significance (*P* < 0.05) of differences between the two groups was assessed using paired Student's* t*-test. All values are expressed as the mean ± SD.

## 3. Results

**Figure 2:**
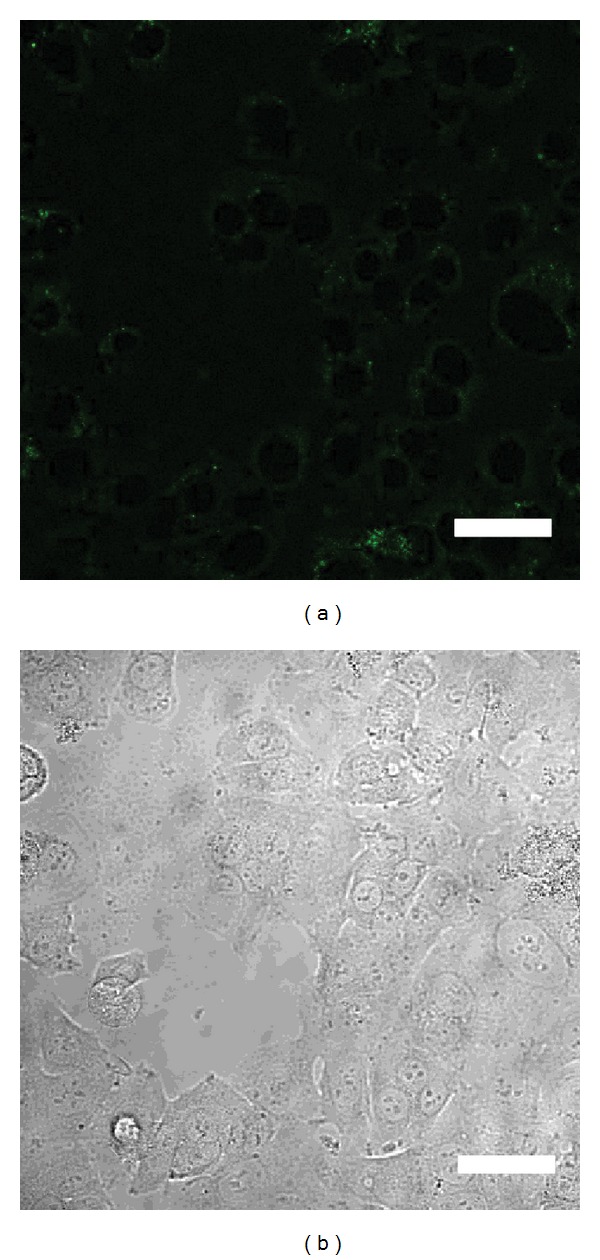
(a) GFP-positive cells after electroporation with 0.005% D-glucosamine-containing buffer. (b) Phase contrast image of area A. Scale bar = 20 *μ*m.

**Figure 3:**
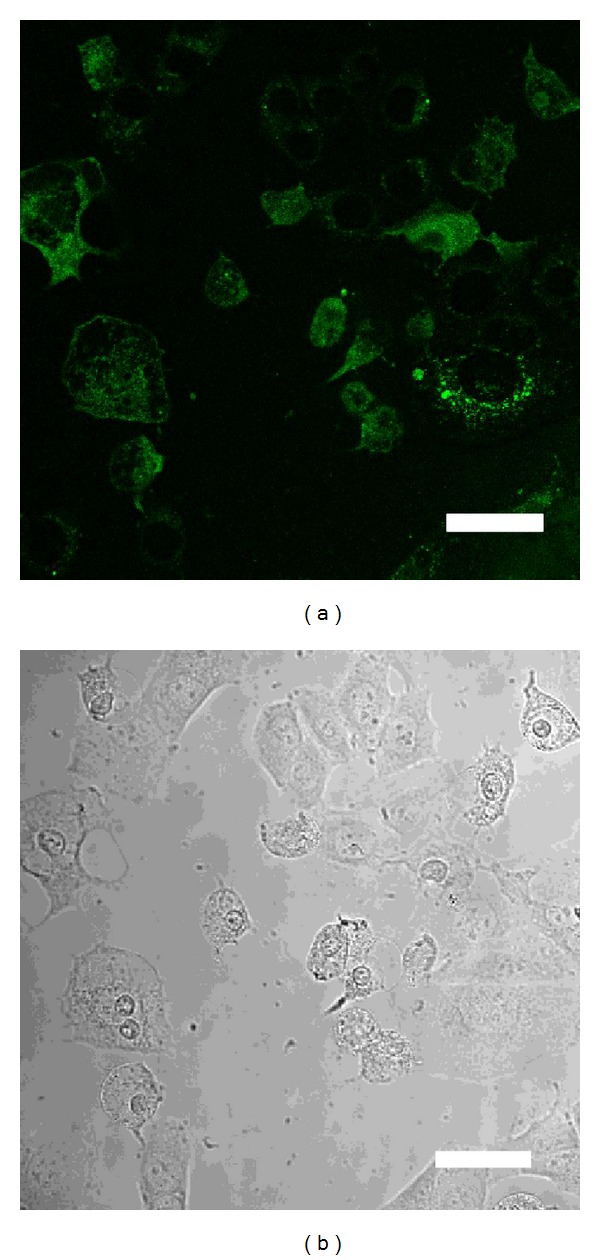
(a) GFP-positive cells after electroporation without 0.005% D-glucosamine-containing buffer. (b) Phase contrast image of area A. Scale bar = 20 *μ*m.

No necrotic cells were observed after electroporation treatment. The percentage of GFP-positive cells was 86.4 ± 4.3% (Figures 2(a) and 2(b)) following electroporation with 0.005% D-glucosamine-containing buffer and 48.7 ± 2.9% (Figures 3(a) and 3(b)) following that without D-glucosamine-containing buffer. The transfection efficiency increased approximately 38%. The percentage between the two groups was significantly different (*P* < 0.01).

## 4. Discussion

This is the first study to observe the stability and protection of the osteoblast membrane against electric stress and genes against lysosomal attack during electroporation with D-glucosamine application. This function of D-glucosamine is relevant for cell biology and biological applications, such as gene therapy.

D-Glucosamine is used as an effective medicament in various fields of medicine and dentistry. For example, it is an attractive candidate for adjunctive therapy in patients with arthritis [7]. D-Glucosamine also has a significant antipain effect in patients with osteoarthritis, a disease with low expectations on the value of treatment [8, 9]. The membrane absorption of D-glucosamine is the primary mechanism of quantum dot (QD) transport into cells (unpublished data). A dramatic increase in the cellular uptake of QDs via attachment with the cell membrane is induced by a positive charge and biocompatibility of conjugated D-glucosamine. This phenomenon was confirmed in control experiments, which clearly indicated that nonconjugated QDs have difficulty entering cells. Another interesting finding is the escape of QDs from lysosomes inside cells, which was confirmed with the observation of merged fluorescence of both QDs and lysosomes. The significant increase in transfection efficiency observed in the present study using D-glucosamine was likely produced by the same mechanism as that underlying the observation of stability and protection of intracellularly distributed QDs following D-glucosamine application. This proton sponge hypothesis, while not definitively proven, has been invoked to explain the relatively high transfection efficiency of other proton-sponge-type materials, such as lipopolyamines [10, 11], PAMAM dendrimers [12], and various imidazole-containing polymers [13–15]. The original hypothesis proposed that PEI buffering in lysosomes induced osmotic rupture and subsequent escape [16]. Although the proton sponge hypothesis based on their findings of a lack of lysosomal involvement is challenged in polyethylenimine- (PEI-) mediated gene transfer, a version of this hypothesis, whereby PEI buffering induces osmotic rupture in endosomes prior to fusion with lysosomes [10, 17], is consistent with the findings of Godbey et al. [18]. Although the pH value of D-glucosamine hydrochloride is acidic (3.5–4.5), the present D-glucosamine solution was used after neutralization. The concentration of endosomal chloride ions originated from D-glucosamine hydrochloride leads to osmotic rupture in endosomes [10, 16], which involves the escape of plasmid vectors from endosomes and lysosomes. The newly proven polycationic function of D-glucosamine through the adsorption to cell membrane and accumulation into cytoplasm (the proton sponge hypothesis: escape from the degradative lysosomal trafficking pathway) is useful and meaningful for both cell biology and clinical applications.

## 5. Conclusion

This is the first study to investigate the stability and protection of the osteoblast membrane against electric stress and genes against lysosomal attack during electroporation with D-glucosamine application. The newly proven polycationic function of D-glucosamine (the proton sponge hypothesis: escape from the degradative lysosomal trafficking pathway) is useful and meaningful for both cell biology and clinical applications.
